# Amperometric Microsensors Monitoring Glutamate-Evoked In Situ Responses of Nitric Oxide and Carbon Monoxide from Live Human Neuroblastoma Cells

**DOI:** 10.3390/s17071661

**Published:** 2017-07-19

**Authors:** Yejin Ha, Chaejeong Heo, Juhyun Woo, Hyunwoo Ryu, Youngmi Lee, Minah Suh

**Affiliations:** 1 Department of Chemistry and Nano Science, Ewha Womans University, Seoul 03760, Korea; hayjej1@hanmail.net; 2 Center for Neuroscience Imaging Research, Institute for Basic Science (IBS), Suwon 16419, Korea; neuroheo@gmail.com (C.H.); woojuinkr@gmail.com (J.W.); a1sw3@nate.com (H.R.); 3 Department of Biomedical Engineering, Sungkyunkwan University (SKKU), Suwon 16419, Korea; 4 Samsung Advanced Institute of Health Science and Technology (SAIHST), Sungkyunkwan University (SKKU), Suwon 16419, Korea

**Keywords:** amperometric sensor, nitric oxide, carbon monoxide, neuroblastoma cells, glutamate stimulation

## Abstract

In the brain, nitric oxide (NO) and carbon monoxide (CO) are important signaling gases which have multifaceted roles, such as neurotransmitters, neuromodulators, and vasodilators. Even though it is difficult to measure NO and CO in a living system due to their high diffusibility and extremely low release levels, electrochemical sensors are promising tools to measure in vivo and in vitro NO and CO gases. In this paper, using amperometric dual and septuple NO/CO microsensors, real-time NO and CO changes evoked by glutamate were monitored simultaneously for human neuroblastoma (SH-SY5Y) cells. In cultures, the cells were differentiated and matured into functional neurons by retinoic acid and brain-derived neurotrophic factor. When glutamate was administrated to the cells, both NO and CO increases and subsequent decreases returning to the basal levels were observed with a dual NO/CO microsensor. In order to facilitate sensor’s measurement, a flower-type septuple NO/CO microsensor was newly developed and confirmed in terms of the sensitivity and selectivity. The septuple microsensor was employed for the measurements of NO and CO changes as a function of distances from the position of glutamate injection. Our sensor measurements revealed that only functionally differentiated cells responded to glutamate and released NO and CO.

## 1. Introduction

Nitric oxide (NO) and carbon monoxide (CO) are multifunctional signaling gases in brain. They are tightly involved in various biological mechanisms such as neurotransmission, neuromodulation, vasodilation, and inflammation, etc. [1,2,3,4]. NO and CO are endogenously generated by enzymes, nitric oxide synthase (NOS), and heme oxygenase (HO), respectively [1,2,3]. It has been reported that among several isoforms of these enzymes—iNOS (inducible form) and nNOS (neuronal form); and HO-1 (inducible form) and HO-2 (constitutive form)—are associated with various types of neuronal cells [2,5,6,7]. Glutamate is one of major excitatory neurotransmitters in the nervous system [8,9]. Synaptically released glutamate activates the post-synaptic *N*-methyl-D-aspartate (NMDA) receptors, mediating signal transmission in the neural system. One of many effects of glutamate-evoked neural responses is the upregulation of NOS for releasing NO [10,11,12]. Excessive amounts of glutamate may induce neurotoxicity, which causes cell death [8,9,13], therefore NO and CO may play roles in glutamate-induced neurotoxicity and/or neuroprotection [1,8,13,14,15].

Although studying NO and CO is important due to their significant roles, several obstacles make it difficult in living biological systems. These gases are produced in minute concentrations and are easily oxidized or react with other molecules [16,17]. Due to these difficulties, indirect methods such as post-mortal analysis of the generating enzymes and measurements of the oxidation products have been used to study NO and CO [18,19,20]. However, direct and real-time measurements are important to reveal their dynamic role in physiological events. Electrochemical methods suggest possible approaches to real-time measurements. The fast response time and low detection limit of electrochemical sensors make it possible to analyze reactive analytes such as NO and CO [21,22]. Moreover, in electrochemical analysis, only a small amount of sample solutions is needed. Also, the damage on specimens can be minimized due to no need of dyes or other chemical agents [22]. Thus, electrochemical sensors have been developed and used for in vivo and in vitro studies on NO and CO. For NO, various electrode materials—such as novel metals; transition metal oxides; carbon nanomaterials; and membranes including Nafion, polytetrafluoroethylene (PTFE), and chitosan—have been adopted for selective NO detections [23,24,25]. The electrochemical NO sensors have been widely applied to rodent brains, livers, kidneys, human, and animal tissues for biological research [24,25]. Contrastively, only a few studies have been reported for electrochemical CO sensors aiming at biological applications due to selectivity problems [26,27]. Our group has developed NO/CO dual sensors for simultaneous NO and CO detections, and applied to in vivo and in vitro studies on rat kidneys and rat brains [28,29,30].

In this work, high performance NO/CO dual [30] and new septuple microsensors were used for monitoring real-time NO and CO changes during glutamate-evoked neuronal activation in vitro. NO and CO were measured in a monolayer of a differentiated SH-SY5Y, which is a widely used dopaminergic neuronal cell type for in vitro model [31]. SH-SY5Y cell was differentiated into functional neuronal cells by retinoic acid (RA) and brain-derived neurotrophic factors (BDNF) [32]. The real-time responses of NO and CO in the glutamate-evoked cells were recorded and compared with those of the non-differentiated cells and a medium without cells as control groups.

## 2. Materials and Methods

### 2.1. Preparations of Dual and Septuple NO/CO Sensors

Amperometric dual and septuple NO/CO microsensors were fabricated based on a previous study [30]. Briefly, a theta type glass capillary (diameter = 1.5 mm, World Precision Instruments, Inc., Sarasota, FL, USA) was thermally sealed with two individually inserted platinum (Pt) wires, which were 76 and 50 µm diameters (Sigma-Aldrich, St. Louis, MO, USA, and Good Fellow, Coraopolis, PA, USA) (working electrode 1 (WE1) and 2 (WE2), respectively), and the wires were electrically connected to copper (Cu) wires (Alpha Wire, Elizabeth, NJ, USA). Pt disks were exposed by grounding the glass capillary on sand paper and diamond film. WE1 and 2 were etched to make micropores at 3 V ac voltage (60 Hz) in 1.2 M CaCl_2_ (Sigma-Aldrich) solution (water:acetone = 2:1 (v:v), Sigma-Aldrich) for 10 s and 2 s, respectively. Then, WE1 was electrodeposited with Au particles in 5 wt % HAuCl_4_ (Alfa Aesar, Ward Hill, MA, USA) and 0.3 M NaCl (Sigma-Aldrich) solution at −0.1 V for 30 s. A thin layer of Pt black was electrodeposited on WE2 with a platinizing solution (YSI Inc., Yellow Springs, OH, USA) at −0.1 V for 5 s. Then, the surfaces of WE1 and WE2 were covered with fluorinated xerogel solution and dried overnight [30]. The compositions of fluorinated xerogel solution are 14.5 μL of heptadecafluoro-1,1,2,2-tetrahydrodecyl trimethoxysilane, 18 μL of methyltrimethoxysilane, 10 μL of 0.5 M HCl, 160 μL of water, and 727.3 μL of ethanol. WE1 and WE2 were polarized at +0.2 V and +0.75 V, respectively, for at least 3 h, and calibrated in deaerated phosphate buffered saline (PBS, pH 7.4, Thermo Fisher Scientific, Waltham, MA, USA) solutions by successive injections of NO (1.91 mM, Dong-A Gas Co., Seoul, Korea) and CO (0.9 mM, Dong-A Gas Co.) saturated PBS solutions. The calibrations were conducted before and after in vitro measurements. In fact, WE1 and WE2 selectively responded to CO and NO, respectively.

All of these processes were repeated with a flower-type glass capillary (World Precision Instruments, Inc.) possessing seven compartments in a single body (diameter = 2.8 mm). Four 76- and three 50-µm diameter Pt wires were placed in the capillary alternately, and modified in the same way as the WE1 and WE2 in the dual sensor, respectively. To test the reproducibility of the electrodes, cyclic voltammograms were obtained with seven electrodes in septuple sensors before and after the metal deposition in 10 mM Ru(NH_3_)_6_Cl_3_ and 0.1 M KNO_3_ solution between 0.15 V and −0.4 V (vs. Ag/AgCl) with a scan rate of 10 mV s^−1^. For all of electrochemical experiments, Ag/AgCl reference electrodes (CH Instruments, Inc., Austin, TX, USA) and CHI 1040C multi-potentiostat (CH Instruments, Inc.) were used.

### 2.2. Cell Culture and Differentiation of SH-SY5Y Cell Line

Cell culture and differentiation processes of SH-SY5Y cell line (ATCC, Manassas, VA, USA, passages 10–30) followed a previous research [32]. A mixture of Dulbecco’s modified Eagle’s medium (DMEM, Thermo Fisher Scientific) with 10% (v/v) fetal bovine serum (FBS, Thermo Fisher Scientific) and 1% (v/v) penicillin streptomycin (Thermo Fisher Scientific) was used as a culture medium. For harvesting of cells, the medium was removed and the cells were detached by 2 mL of cell dissociation solution (Sigma-Aldrich) from a T75 flask (75 cm^2^, Thermo Fisher Scientific) bottom and collected in a conical tube with 10 mL PBS solution (pH 7.4, Thermo Fisher Scientific). After centrifugation at 1200 rpm for 5 min, the cell pellet was mixed with 2 mL of the culture medium. The 10 µL of solution was mixed with a same volume of trypan blue (Sigma-Aldrich) and cells in the solution were counted by hemocytometer. The 3 × 10^5^ cells were dispersed in a T75 flask with 25 mL of a fresh cell culture medium mixture in subculture. Cells were housed in a 37 °C incubator filled with a mixed atmosphere of 95% air and 5% CO_2_ (Dong-A Gas Co.).

For differentiation of the cells, the cells were treated 10 µM of retinoic acid (RA, Sigma-Aldrich) on Day 2, a day after the cells were seeded on a 12-well plate (Sumitomo Bakelite Co., LTD., Tokyo, Japan), and changed again with a fresh medium with 10 µM RA on Day 4. On Day 6, the medium was changed with a serum-free medium (i.e., a mixture of DMEM and 1% penicillin streptomycin) having 50 ng mL^−1^ brain-derived neurotrophic factor (BDNF, Sigma-Aldrich) instead of RA. Then, the medium was changed with a fresh serum-free medium with 50 ng mL^−1^ BDNF on Days 8 and 10. On Day 12, two days after the last BDNF treatment, the cells were used for NO and CO measurements. All processes were repeated with SH-SY5Y cells in only corresponding medium mixtures without RA and BDNF for a non-differentiated control group.

### 2.3. NO and CO In Situ Measurements on Neuronal Cells

**Figure 1:**
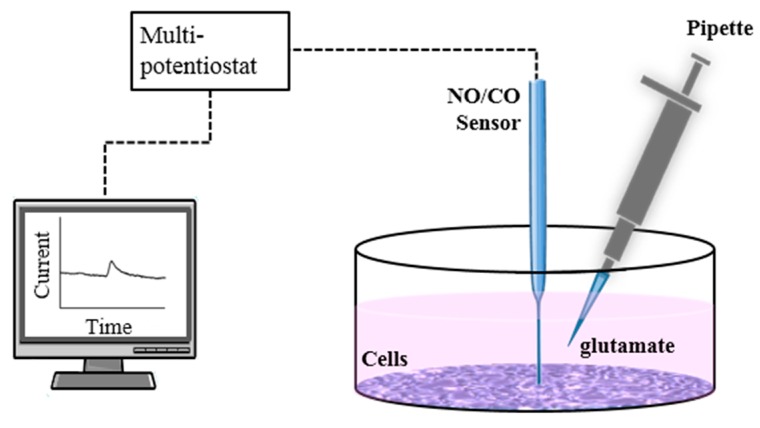
The experimental setup for real-time NO and CO measurements for functional human neuroblastoma cells with a glutamate injection. A vertical distance between the end plane of a sensor and cell monolayer surface is approximately 10 μm. NO/CO sensor in this figure represent either dual or septuple sensors.

Two types of experiments were performed for NO and CO measurements. Two hours prior to the experiments, the cell medium was changed to 2 mL of fresh serum-free medium which was a mixture of DMEM and 1% penicillin streptomycin. Firstly, the dual sensor was placed on the differentiated cells in the medium (Figure 1). After the stabilization of the sensor, 50, 100, and 200 µL of 1 µM; 100 µL and 200 µL of 20 µM; and 100 µL of 100 µM glutamate (Sigma-Aldrich) solutions were injected into the medium near the sensor every 200 s to reach final glutamate concentrations of 25 nM, 50 nM, 100 nM, 1 µM, 2 µM, and 5 µM, and the signal responses were recorded during the process. It was repeated with non-differentiated SH-SY5Y cells and a serum-free medium. Secondly, the septuple sensor was placed on the differentiated cells, and 100 µL of 1 µM glutamate was injected to reach 50 nM in 2 mL medium with a micro-pipette once the sensor current became stabilized (Figure 1). This process was repeated in a serum-free medium without containing cells as a control group. For all of the experiments, the gaps between the sensor surfaces and cells (or bottom of the plate without cells) were approximately 10 µm controlled by micro-manipulators (Narishige, Tokyo, Japan and Mitutoyo, Kanagawa, Japan), and the injection speed of glutamate was approximately 20 µL s^−1^. In fact, the sensors were placed slightly touching the dish bottom where no cells were found, and then retracted by 10 µm using a micro-manipulator, followed by the lateral movement to the targeting location with plenty of cells. WE1 of the dual sensor and electrode no. 1, 3, 5, 7 of the septuple sensor were polarized at +0.2 V, and WE2 of the dual sensor and electrode no. 2, 4, 6 of the septuple sensor were polarized at +0.75 V during the experiments. Optical microscopic images of the dual and septuple microsensor end planes are shown in Figure S1. All measurements were conducted in a live incubating chamber maintaining 37 °C and 5% CO_2_.

## 3. Results and Discussion

Electrodeposited Au and Pt layers enhanced sensitivities to NO and CO. The applied potentials to the WEs were optimized for selective oxidations of the targeted gases. The gas permeable fluorinated xerogel membrane covering the surface of the sensor provided high selectivity against common biological interferents, such as ascorbic acid, dopamine, and uric acid. The WE geometry, with recessed pores filled with Au or Pt particles, minimized contamination or damage of the electrode surface. The details of a NO/CO dual sensor were described in the previous study [30]. The NO/CO dual and septuple sensors measured NO and CO simultaneously at two and seven electrodes, respectively, and showed high sensitivity and selectivity. The septuple sensor consisted of three NO electrodes (no. 2, 4, and 6) and four CO electrodes (no. 1, 3, 5, and 7). As a reproducibility test of the electrodes in septuple sensors, cyclic voltammograms of NO and CO electrodes were obtained in 10 mM Ru(NH_3_)_6_Cl_3_ + 0.1 M KNO_3_ solution before and after the metal depositions (Figure S2). The steady state currents (*i*_ss_) and capacitive currents were higher at Pt and Au deposited electrodes (NO and CO electrodes, respectively) than those of the bare electrodes. The average *i*_ss_ of metal deposited NO and CO electrodes were 102.4 ± 3.7 nA (*n* = 6) and 176.4 ± 22.4 nA (*n* = 8), respectively. Although larger distributions of the metal deposited electrodes were observed compared to the bare electrodes (*i*_ss_; 89.1 ± 1.1 nA (*n* = 6) and 143.5 ± 2.3 nA (*n* = 8) for NO and CO electrodes, respectively) due to the enhanced surface area by deposited metal particles, they still show high reproducibility for electrodes in the septuple sensors. Figures S3 and S4 show the simultaneously obtained dynamic response curves and averaged calibration curves of the seven electrodes to NO and CO, respectively. The NO electrodes and CO electrodes exhibited the current increases only responding to NO and CO, respectively. The detection limits were ~6.0 nM to NO at NO electrodes and ~180 nM to CO at CO electrodes (*S/N* = 3). Both NO and CO electrodes in the septuple sensor showed high selectivity to NO and CO over common biological oxidizable interfering species as shown in Figure S5.

**Figure 2:**
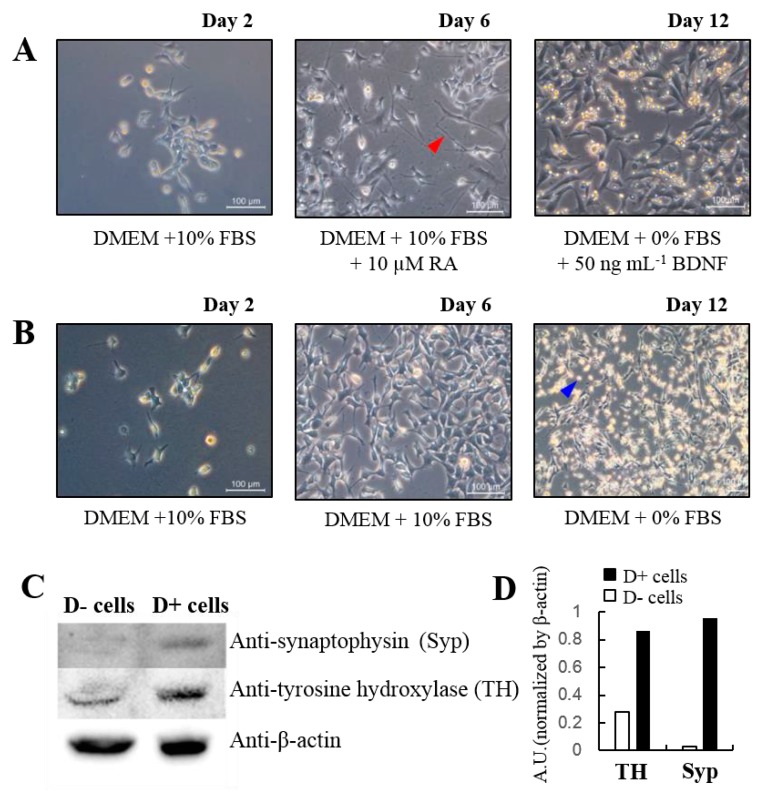
Phase contrast images of cultured cells (**A**) with and (**B**) without cell differentiation factors, RA and BDNF. Western blot analysis (**C**) with differentiated, D+, and non-differentiated, D−, cells and the graph (**D**) showed the quantified protein levels. Dulbecco’s modified Eagle’s medium (DMEM), fetal bovine serum (FBS), retinoic acid (RA), and brain derived neurotrophic factor (BDNF).

To investigate the relationship between NO/CO and glutamate-evoked neuronal responses, SH-SY5Y cells were differentiated into neuronal cells that reflected characteristics of functional dopaminergic neuron cells [32]. The images of the differentiated (denoted as D+) and non-differentiated (denoted as D−) cells were showed in Figure 2. The SH-SY5Y cells were well attached and grown in culture dish (Day 2 in Figure 2A,B). The D+ cells were cultured under RA treatment for five days, which can promote cells’ differentiation into functional neurons with enhanced synaptic process and channel development. The enhanced synaptic process and channel development were investigated at protein levels by Western blotting (Figure 2C). Western blotting data indicates that tyrosine hydroxylase (TH), a specific enzyme in the dopaminergic cells, and synaptophysin, a synaptic marker protein in functional neuron, were clearly detected from D+ cells [33]. The graph showed significant difference these protein levels between D+ and D− cells with normalized by beta-actin amount (Figure 2D). In addition to these protein analyses, the Day 6 image in Figure 2A showed that differentiated cells possess the long processing neurite, a typical neuronal indicator as marked by red arrow head. The neurites were well developed and connected between each neurons in D+ cells, whereas no clear neurites were found in D− cells (Figure 2B). More specifically, D− cells showed well defined proliferation under the serum (10% FBS) but less neurite development than D+ cells. To further differentiate cells into stable functional neurons, we treated cells with BDNF for seven days [32]. Under BDNF, cells became healthier and more stable neuronal cells in an image at Day 12 in Figure 2A. While D− cells in serum-free media condition showed many floating cells indicated by the blue arrow head in Figure 2B. Also, D− cells without serum tended to detach in the culture dish.

**Figure 3:**
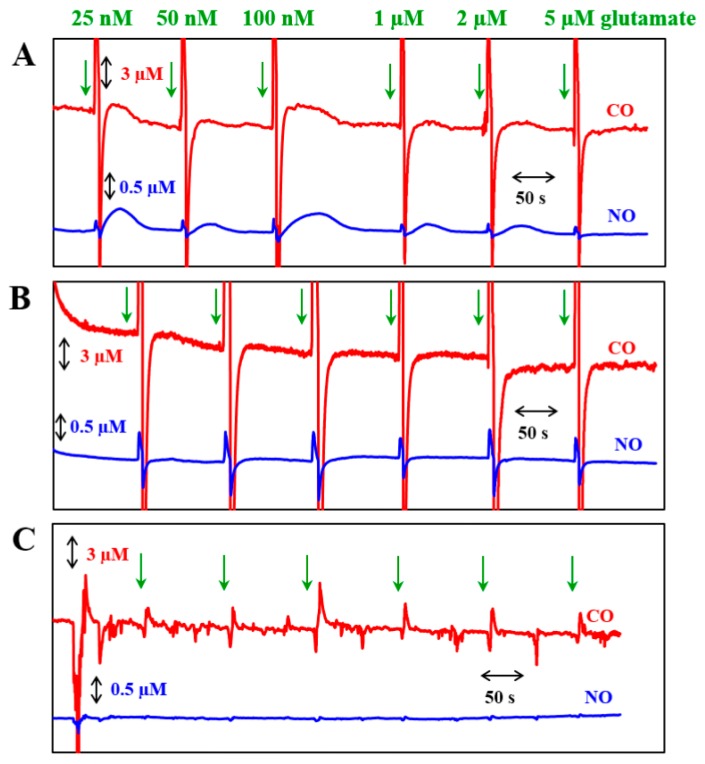
NO and CO concentration changes responding to sequential glutamate injections (time points are marked with green arrows), monitored for (**A**) differentiated cells; (**B**) non-differentiated cells; and (**C**) only serum-free medium with a dual NO/CO microsensor.

NO and CO measurements were conducted with the fabricated dual and septuple sensors for D+ and D− groups of cells on Day 12. The cells were rinsed via changing the fresh medium before the NO and CO measurements therefore any floating cells were cleared. Since the cell diameter is estimated to be 19.3 ± 4.4 µm, each WE (76 μm in diameter for WE1 and 50 μm in diameter for WE2) of the sensors seemingly detects NO and CO generated from several cells near the WE. Firstly, the dual-type NO/CO sensor was used to compare NO and CO responses to glutamate administration of three different samples: D+ cells (Figure 3A), D− cells (Figure 3B), and only medium without cells (Figure 3C). When 25 nM, 50 nM, 100 nM, 1 µM, 2 µM, and 5 µM of glutamate solutions were loaded sequentially by an injection pipette, NO and CO changes were observed only with D+ cells, but there were no significant responses with D− cells and a medium without cells. The NO and CO measurements with D+ cells (Figure 3A) showed gradual increases and decreases following glutamate injection. Note that sharp peaks in Figure 3 were noises caused by the handling action of injections. For each glutamate injection, ΔNO (or ΔCO) was calculated based on the following equation.

ΔNO(or ΔCO) = NO(or CO)_max_ − NO(or CO)_base_ (1)

where NO(or CO)_base_ and NO(or CO)_max_ are average concentration values of NO or CO for 5 s in steady-state before and around peaks after each glutamate injection, respectively.

ΔNO was recorded as 196.2 ± 118.7 nM, and ΔCO was 806.0 ± 619.7 nM. ΔNO and ΔCO were not dependent to glutamate concentrations but shared similar trends, i.e., higher ΔNO was accompanied with higher ΔCO. The required times to return to the basal levels after the glutamate injection were 143.7 ± 34.7 s and 140.5 ± 33.1 s for NO and CO, respectively. The required times to reach the peaks after the glutamate injections were 65.4 ± 20.3 s and 57.8 ± 12.5 s for NO and CO, respectively. CO was generated and peaked earlier than NO. As aforementioned, the spikes observed at both WE1 and WE2 for all of three samples with glutamate injections might be artifacts due to sudden flow changes from adding solutions.

**Figure 4:**
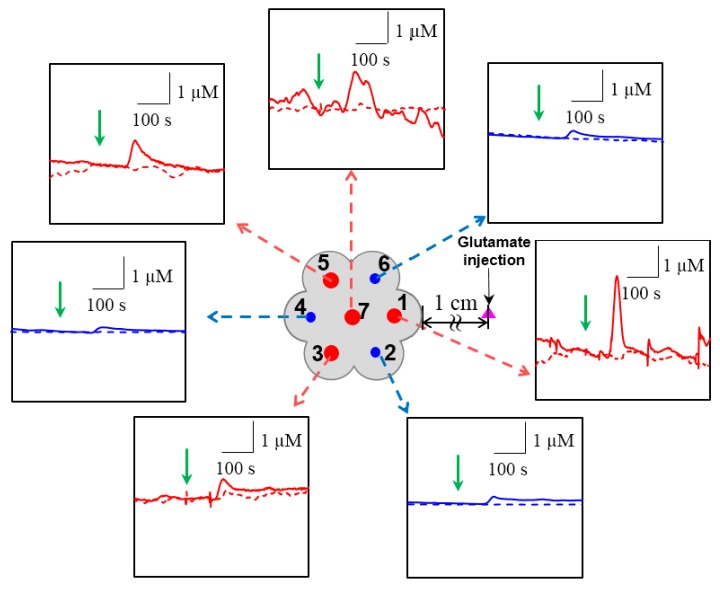
The concentration changes of NO (blue, locations 2, 4, 6) and CO (red, locations 1, 3, 5, 7) responding to 50 nM glutamate administration (marked with green arrows), measured with a septuple NO/CO sensor for differentiated cells (solid lines) and in only medium without cells (dashed lines). The glutamate injection site is marked with a pink triangle.

Secondly, a septuple sensor consisting of seven electrodes (three for NO and four for CO measurements) was used to monitor NO and CO levels responding to one time glutamate injection for D+ cells and a medium without cells. Figure 4 illustrates NO and CO changes responding to the injection of 50 nM glutamate. Both NO and CO increased alongside with the glutamate injection into D+ cells (solid lines in Figure 4), whereas there were no noticeable changes in the medium only (dashed lines in Figure 4). The averaged ΔNO and ΔCO were 155.1 ± 37.4 nM and 1.285 ± 0.704 µM, respectively. The location dependent ΔNO and ΔCO were shown in Figure S6A,B, respectively. Locations 2 and 6 which were positioned at the same relative distance from the injection site (marked with a pink triangle in Figure 4) exhibited similar ΔNO values. At location 4 with a longer relative distance from the injection site, smaller ΔNO was measured compared to the ones at locations 2 and 6. Likewise, ΔCO showed the same tendency as ΔNO: a closer location to the injection site was accompanied by a higher ΔCO. In Figure 4, green arrows indicate the moment when glutamate was injected into the medium. In this experiment, the glutamate injection was performed approximately 1 cm apart from the sensor to minimize noises caused by the injection itself. Thus, time gaps (Δ*t*) were observed between the glutamate injection and onset times. The location dependent Δ*t* for NO (Δ*t_NO_*) and CO (Δ*t_CO_*) were shown in Figure S6C,D, respectively. The observed Δ*t_NO_* and Δ*t_CO_* also depended on the actual distance between the injection and sensing locations, i.e., closer electrodes to the glutamate injection point generated earlier NO and CO onsets. Also, the average of Δ*t_NO_* (33.2 ± 3.4 s) took longer than that of Δ*t_CO_* (28.4 ± 4.6 s). From the results which show faster responses of CO compared to those of NO in spite of relatively slow diffusion rate [34], it is assumed that releasing rate of CO might be faster than NO in the cells.

In terms of the sensor size, the end plane cross-sectional dimension of a septuple sensor including glass sheath was 40.3 times larger than that of a dual-type sensor. It might affect the concentrations and change patterns of NO and CO. In fact, NO and CO peaks observed with a bulky septuple sensor were sharper in shape and greater in magnitude than the ones measured with a rather small dual-type sensor (Figure 4 vs. Figure 3). NO and CO generated from the cells could be trapped within a gap (10 μm) between the large septuple sensor end plane and the cells, which possibly caused faster and higher concentration increases. The dual-type sensor could also cause the trapping effect to a lesser degree. This trapping effect presumably resulted in a quite high gas concentration which could be advantageous for monitoring these gases being generated at low levels. In addition, since the septuple sensor measured NO and CO at seven different sites simultaneously, it provided location-dependent information of NO and CO gases generated from the living cells more efficiently. We report the real-time simultaneous measurements of NO and CO generated from the activated neuronal cells in vitro with a developed high sensitivity electrochemical sensor. Our sensor can serve as an effective tool to study the gaseous signaling molecules’ in situ dynamic and its physiological role in living biological systems in functional neuroscience studies.

## 4. Conclusions

NO and CO were measured with NO/CO dual and septuple sensors for neuronal cells. WE1 for CO detection in dual and septuple sensors was electrodeposited with Au particles and covered with fluorinated xerogel membrane to obtain high sensitivity and selectivity toward CO. The optimized potential, +0.2 V vs. Ag/AgCl, was applied to the electrodes for the selective oxidation of CO against NO. For WE2 detecting NO, thin layers of Pt were deposited with an amperometric method, and silanized with fluorinated xerogel membrane to enhance sensitivity and selectivity to NO over interfering species including CO +0.75 V vs. Ag/AgCl was applied to WE2 for NO oxidation. The fabricated dual and septuple sensors showed high sensitivity to NO and CO, and high selectivity over common biological interfering species such as ascorbic acid or dopamine. SH-SY5Y cells were cultured with RA and BDNF to enhance its neuronal phenotype which was functionally active. Differentiation of the cells was confirmed with neurite development and protein levels by anti-synaptophysin antibody and anti-TH antibody performed with Western blot analysis. With the dual-type sensor, NO and CO were measured for glutamate-evoked responses in differentiated functional neurons and non-differentiated cells; and in a medium without cells by loading various concentrations of glutamate solutions. Increases of NO and CO levels were observed only with differentiated cells. NO and CO measurements revealed that there was no glutamate concentration dependency with NO and CO responses. Secondly, the septuple sensor was used to monitor NO and CO changes of differentiated cells responding to the addition of 50 nM glutamate, which was compared with the measurements in a medium without cells. NO and CO increased only with activated neurons, and their onset times and concentrations depended on the distance between the electrode locations and glutamate injection site.

## Supplementary Materials

The Supplementary Materials are available online at http://www.mdpi.com/1424-8220/17/7/1661/s1. Figure S1: Optical microscopic images of the sensors. Top views of (A) dual and (B) septuple NO/CO microsensors. WE1 in A and Electrode No. 1, 3, 5 and 7 in B were 76 µm in diameter, and WE2 in A and Electrode No. 2, 4 and 6 in B were 50 µm in diameter. The average distance between NO and CO electrodes in the dual sensor is 84.34 ± 10.63 µm (*n* = 8). The average distance between electrodes in the septuple sensor is 307.36 ± 26.33 µm (*n* = 12). The distances between electrodes are measured as lengths between centers of two adjacent disks. Figure S2: Representative cyclic voltammograms of (A) metal deposited and (B) bare 50-µm diameter NO electrodes (blue) and 76-µm diameter CO electrodes (red) in 10 mM Ru(NH_3_)_6_Cl_3_ + 0.1 M KNO_3_ aqueous solution between 0.15 V and −0.4 V (vs. Ag/AgCl) with scan rate of 10 mV·s^−1^. Figure S3: (A–G) Dynamic response curves of the seven electrodes in a septuple sensor to stepwise increase of NO concentration. The injection of a NO standard solution caused the current fluctuations at CO electrodes, which were returned to the background currents eventually. (H) Calibration curves showing the averaged currents and error bars, corresponding to the dynamic response curves of the NO electrodes (No. 2, 4, and 6: solid line) and the CO electrodes (No. 1, 3, 5, and 7: dashed line). The averaged sensitivity of the NO electrodes was 46.0 ± 3.21 pA·µM^−1^ with a high linearity (R^2^ = 0.998). Figure S4: (A–G) Dynamic response curves of the seven electrodes in a septuple sensor to stepwise increase of CO concentration. The injection of a CO standard solution caused the current fluctuations at NO electrodes, which were returned to the background currents eventually. (H) Calibration curves showing the averaged currents and error bars, corresponding to the dynamic response curves of the NO electrodes (No. 2, 4, and 6: dashed line) and the CO electrodes (No. 1, 3, 5, and 7: solid line). The averaged sensitivity of the CO electrodes was 62.0 ± 3.53 pA·µM^−1^ with a high linearity (R^2^ = 0.998). Figure S5: Dynamic current responses of the seven electrodes in a septuple sensor to the sequential additions of 50 µM acetaminophen (AP), 50 µM ascorbic acid (AA), 10 µM dopamine (DA), 50 µM uric acid (UA), and 50 µM nitrite, in order. Although the currents were slightly increased with the additions of AP, AA, and DA particularly at the electrodes No. 1, 3, and 5, all the electrodes showed reasonable selectivity to CO and NO, with selectivity coefficients (log $K_{\mathrm{CO}\left(\mathrm{or}\;\mathrm{NO}\right),\;i}^{\mathrm{amp}}$ < −2, where log $K_{\mathrm{CO}\left(\mathrm{or}\;\mathrm{NO}\right),\;i}^{\mathrm{amp}}$ = log$\left(\frac{\mathrm{sensitivity}\;\mathrm{to}\;i}{\mathrm{sensitivity}\;\mathrm{to}\;\mathrm{CO}\left(\mathrm{or}\;\mathrm{NO}\right)}\right)$ and *i* = interfering species. The injection volume for all of the interferents was 30 µL. Figure S6: Plots of (A) ΔNO, (B) ΔCO, (C) ΔtNO, and (D) ΔtCO depending on the relative distances between the glutamate injection site and the location of each electrode in Figure 4. For NO electrodes (No. 2, 4 and 6), shorter distances between the glutamate injection site and electrode No. 2 and 6 were denoted as D1, and the longer distance between the glutamate injection site and electrode No. 4 was denoted as D2. In the same principle, the relative distance between the glutamate injection site and CO electrodes (No. 1, 3, 5 and 7) were denoted as D1, D2 and D3. The numbers on the graphs were matched with the electrode numbers in Figure 4, indicating the electrode locations.
